# Knowledge of COVID-19 and Its Relationship with Preventive Behaviors and Vaccination among Adults in Northern Thailand’s Community

**DOI:** 10.3390/ijerph19031521

**Published:** 2022-01-28

**Authors:** Tharadon Pothisa, Parichat Ong-Artborirak, Katekaew Seangpraw, Prakasit Tonchoy, Supakan Kantow, Nisarat Auttama, Sorawit Boonyathee, Monchanok Choowanthanapakorn, Sasivimol Bootsikeaw, Pitakpong Panta, Dech Dokpuang

**Affiliations:** 1 School of Medicine, University of Phayao, Phayao 56000, Thailand; tharadon.po@up.ac.th (T.P.); sorawit.bo@up.ac.th (S.B.); 2 Faculty of Public Health, Chiang Mai University, Chiang Mai 50200, Thailand; parichat.ong@cmu.ac.th; 3 School of Public Health, University of Phayao, Phayao 56000, Thailand; prakasit.to@up.ac.th (P.T.); supakan.ka@up.ac.th (S.K.); nisarat.au@up.ac.th (N.A.); monchanok.ch@up.ac.th (M.C.); sasivimol.bo@up.ac.th (S.B.); 4 School of Nursing, University of Phayao, Phayao 56000, Thailand; pitakpong.pa@up.ac.th; 5 School of Allied Health Sciences, University of Phayao, Phayao 56000, Thailand; dech.do@up.ac.th

**Keywords:** knowledge, preventive behaviors, COVID-19 vaccine, adults, northern Thailand

## Abstract

(1) Background: the 2019 coronavirus disease outbreak (COVID-19) has posed a major threat to public health and had a significant impact on all areas of people’s lives. Vaccines against COVID-19 have been developed to control the disease, and an array of personal hygiene measures has been introduced. As a result, information that will support and promote vaccination among populations as well as other health measures against COVID-19 are urgently needed. The goal of this research was to look into the knowledge about COVID-19 and how it relates to preventive behaviors and vaccination among people living in rural areas of northern Thailand. (2) Methods: a cross-sectional study was performed in four upper northern provinces of Thailand. A total of 1524 participants were recruited using the probability sampling technique. Questionnaires were distributed to collect data on general health information, as well as knowledge and preventive behaviors regarding COVID-19. (3) Results: more than half (55.9%) of the participants were female and had not received the COVID-19 vaccine (67.2%). Their mean age was 44.13 years. The majority had moderate COVID-19 knowledge and engaged in preventive behaviors (65.5% and 42.0%, respectively). A linear regression model showed that the COVID-19 knowledge score was related to the COVID-19 preventive behaviors score, with a standardized coefficient of 0.510, after adjusting for age, underlying disease, and body mass index (B = 2.64; 95%CI = 2.42, 2.87). Binary logistic regression revealed that after controlling for age, education, occupation, financial status, and current disease (AOR = 1.87; 95%CI = 1.64–2.13), the score of COVID-19 knowledge was significantly associated with having the COVID-19 vaccine. (4) Discussion: knowledge of COVID-19 is very important for people in rural regions to engage in COVID-19 prevention behaviors and vaccination. Relevant government agencies and health network partners should support proactive education campaigns emphasizing the risk of contracting the disease and its severity in order to promote vaccination against COVID-19 among unvaccinated groups. These campaigns can highlight COVID-19’s positive benefit-risk balance in the short and long term and ensure public safety measures against COVID-19.

## 1. Introduction

The COVID-19 pandemic has affected all populations in different ways, causing sickness and mortality on a global scale. At the same time, it has had a serious impact on our society and economies around the world [1]. The COVID-19 outbreak has overwhelmed healthcare systems in almost every country. For example, since 2020 there have been more than 35 million confirmed cases and more than one million reported deaths [2]. The disease causes serious impacts on health, especially among high-risk populations, including obese persons, smokers, and patients with cancer, chronic kidney disease, heart disease, immunodeficiency states and type 2 diabetes [1,2,3]. The best measures to prevent infection are social distancing, lockdowns, wearing facemasks, and avoiding exposure [2,4]. However, these measures have required many businesses around the world to shut down, resulting in physical and mental health problems, no social interaction among people, and global economic recession [3,4]. The serious consequences associated with COVID-19 have strengthened the need for effective vaccines to control and prevent the spread of the disease [4,5]. Previous studies indicate that individuals are willing to pay up to $290 for the COVID-19 vaccine, while 10–20% of populations refuse to pay for vaccines [4,5].

Using vaccines has been a successful measure for eradicating and preventing infectious diseases for decades [6]. From the perspective of public health, vaccination is the most effective way to prevent the spread of infectious diseases [7]. Previous studies indicate that influenza vaccination is recognized as the most effective method for preventing seasonal influenza and related infectious diseases [8]. In December 2020, several vaccines against COVID-19 were authorized to use among the population to prevent and control infectious disease; and more than 50 vaccines against COVID-19 were developed [9]. Some members of the public have expressed doubts about the rapid development of vaccines in terms of safety and efficacy because there are multiple reported cases of re-infection after receiving the vaccines [10,11]. However, vaccine hesitancy and refusal are significant concerns globally, prompting the World Health Organization (WHO) to declare this uncertainty as being one of the top 10 health threats in 2019 [10,11,12,13]. Despite many doubts, vaccination against COVID-19 is well underway in many countries around the world, including Thailand which planned to start vaccinating its population from February 2021 [14,15].

Currently, there is a third wave of COVID-19 in Thailand, and the number of infected people has increased exponentially, with a cumulative total of 1,523,775 cases since April 2021 [15]. Following the outbreak and severity of COVID-19, the Thai government has made many preparations related to the procurement of vaccines in order to provide Thai people with safety access and effective vaccines [15]. One study suggests that compliance with state and national control measures is very important in preventing infectious diseases [16]. Lessons learned from the SARS outbreak in 2003 suggest that knowledge and understanding of infectious diseases are correlated with the emotion and panic levels of the population, which could complicate efforts to prevent the spread of the disease [17]. A Knowledge, Attitudes, and Practices (KAP) study showed that infectious disease prevention behavior in the context of COVID-19 is associated with knowledge, attitudes, and practices [18]. Hesitation, contrasting views, and a lack of understanding of the efficacy and effects of vaccines have influenced people’s decisions on whether or not to receive vaccines against COVID-19. Similar to previous studies, hesitation and misinformation regarding the vaccines are among the barriers preventing people and communities from gaining immunity against the infection [6,19]. Some studies have suggested that reasons for this hesitation include religious and personal beliefs, safety concerns, and health complications related to the vaccines [20].

Statistics show that in Thailand’s 77 provinces, a total of 33,427,463 vaccines had been administered as of 1 September 2021. A first dose was administered to 24,147,532 people (33.5% of the population), a second dose was administered to 8,684,695 people (12.1% of the population), and a third dose has been administered to 595,236 people (0.8% of the population) [21]. When categorizing vaccination by regions of Thailand, it was found that most of the vaccinated population lives in the central region, including Bangkok and its nearby provinces, whereas populations in the northern region had a low rate of vaccination [21]. As a result, knowledge information to support and promote vaccination among populations, as well as COVID-19 preventive behaviors, are essential. To the best of our knowledge, there are no previous published papers investigating factors related to COVID-19 vaccination among the Thai population. This study aimed to explore the association among personal factors, COVID-19 knowledge, preventive behaviors, and vaccination among people living in rural areas of northern Thailand. The survey of variables associated with COVID-19 vaccination is very important for tailoring appropriate and effective activities to increase vaccination acceptance among the rural Thai population.

## 2. Materials and Methods

Our cross-sectional study, under the Unit of Excellence Project “Health Promotion and Quality of Life”. Data were collected between September–November 2021 in the rural areas of four upper-northern provinces of Thailand. In this study, purposive sampling was employed using the following procedures: (1) We selected provinces in which the COVID-19 outbreak occurred during phase 1–3, and more than 50 cases were reported in the Health Service 1 area in 2021; (2) the study then conducted area cluster sampling by dividing the administrative areas in each province, including Phayao with nine districts, Chiang Mai with 25 districts, Chiang Rai with 18 districts, and Lamphun with eight districts. The sample district areas were obtained using simple random sampling with the lottery technique. The districts areas are Phayao Province, Mueang District; Chiang Mai Province, Mae Rim District; Chiang Rai Province, Mae Chan District; and Lamphun Province, MaeTha District. Then, the participants in the study were selected from each district using simple random sampling. The inclusion criteria were: (a) females and males aged 20 years and over, (b) living in the selected area for at least one year, and (c) voluntarily signed written consent to participate in the study. Persons with cognitive or psychological disorders or gestational diabetes were excluded. Before conducting the research, an announcement was made in the community to recruit 15 research assistants from each sub-district, including three public health scholars and 10 village health volunteers. Cochran’s formula [22] was used to calculate sample size with a 95% confidence level, a proportion of 35%, and a precision of 5% for predicted 30–40% COVID-19 vaccination in northern Thailand during September and October. The sample was increased by 5%, resulting in 385 people being used as samples in the study. A total of 1540 people from four provinces took part in the study. Finally, data from 1524 participants were evaluated, with missing data excluded.

A research meeting in each area was held four hours prior to the data collection day. The meeting was held to clarify the objectives of the study, the data-collection technique using a questionnaire, scheduling interviews, and protecting the rights and privacy of the study participants. The researcher translated the official language into the local language so research assistants would better understand the content. Because the research was performed during the third wave of the COVID-19 pandemic in Thailand, actions had to be authorized by the local government officer and village headman, and prevention measures announced by the government had to be followed during the process of data collection. Data collection was done by liaising with the District Public Health Office in each province before conducting the research in the area. A door-to-door data collection was obtained at the participants’ homes or at a primary care unit for those who visited to receive health services between 9:00 a.m. and 5:00 p.m. or at their convenience. A research assistant interviewed each participant, a procedure that lasted approximately 30–45 min. A small gift was given to each participant for completing the interview.

The questionnaire used to obtain quantitative data was applied from the research and developed to be suitable for research areas in rural areas of northern Thailand. The questionnaire consists of three parts, as follows: Part I: socio-demographic information, including sex, age, marital status, education, occupation, and financial status. Health information, including body mass index (BMI), current disease, smoking, drinking alcohol, exercise, dietary supplements, receiving COVID-19 information, and the COVID-19 vaccine. Part II: COVID-19 knowledge, which was developed to be suitable for the study’s population [16,23]. The questions consisted of: (1) COVID-19 transmission (3 items); (2) COVID-19 symptoms (5 items); and prevention of COVID-19 (4 items)—a total of 12 items. The questions were multiple choice and required the participants to mark the answers as either correct or wrong, with a total score range of 0–12 points. Part III: COVID-19 preventive behavior, which was developed to be suitable for participants living in rural areas [16,23]. There is a total of 15 items, using three levels of the rating scale, including never practice, sometimes practice (3–4 times/week), and regularly practice (more than 5–6 times/week). All questionnaires were applied and developed based on an intensive review of the literature. After completing the first draft of the questionnaire, it was checked for accuracy using item-objective congruence (IOC); and three external experts in their respective fields (Internal Medicine, Behavioral Health, and Public Health) checked the suitability of the content in the questionnaire. Questionnaire scores less than 0.5 were eliminated, answers that scored between 0.5–0.69 were revised based on expert feedback; and answers with a score greater than 0.7 were considered acceptable. The questionnaires were tried out among 30 participants with similar characteristics as those living in the area. A reliability test was performed on Part II of the questionnaire using the Kuder-Richardson formula: KR20 = 0.80. Parts II and III obtained the reliability of the questionnaire analyzed using Cronbach’s alpha coefficients of 0.82 and 0.86.

### Data Analysis

We performed statistical analysis using SPSS version 17, licensed from Chiang Mai University (SPSS Inc., Chicago, IL, USA). The Chi-square test was used to compare the proportion of categorical variables in groups that did and did not receive vaccines. An unpaired *t*-test was used to determine the differences in continuous variables between two groups (the variables were female vs. male, married vs. single/widowed/divorced/separate, sufficient income vs. insufficient income, yes vs. no for getting the vaccine, having a disease, smoking, drinking alcohol, exercising, eating herbs, eating vitamin C, and receiving information about COVID-19), whereas a one-way ANOVA was used to compare at least three groups in terms of age, education, occupation, and BMI. The Pearson correlation coefficient (r) was used to investigate the relationship between COVID-19 knowledge and preventive behaviors. Multiple logistic and linear regression was used to identify COVID-19 knowledge and the sociodemographic variables (sex, age, marital status, education, occupation, financial status, BMI, and current disease) associated with getting the vaccine and COVID-19 preventive behaviors, respectively. The final multivariable model was given, in which all factors were found to be significant. All tests were two-tailed, and statistical significance was defined as *p* < 0.05.

## 3. Results

### 3.1. Characteristics of the Study Participants

The sample characteristics are presented in Table 1. One thousand one hundred and seventy-four participants were enrolled in the study. The average mean age of the overall sample population was 44.13 (Min–Max = 20–89, SD = 16.25). Around 22.7% of participants were above 60 years old. Females represented 55.9% of the study population, while 44.1% were males. A third of the participants (32.8%) had been vaccinated against COVID-19. When asked about the type of COVID vaccine received, the study found that most of those vaccinated had received Sinovac vaccines (70.0%) and AstraZeneca vaccines (30.0%). Approximately half were single (50.9%), one-third of the participants (85.4%) were educated. Most of them had an occupation (86.0%), almost two-thirds (61.8%) were self-sufficient in terms of financial status. Nearly half of the participants had a normal BMI (47.8%), 30.8% had a current disease, and more than half (69.5%) were found to have HT followed by dyslipidemia (DM) (37.3%), and chronic kidney disease (6.8%), respectively. Among the 998 sample participants (65.5%) did not smoke and 914 (60.0%) did not drink alcohol. Furthermore, they consumed herbs such as andrographis and ginger (33.5%) and took vitamin C (13.9%). Most of them received COVID-19 information (67.8%). When analyzing factors related to COVID-19 vaccination, we found that age, education, occupation, financial status, current disease, smoking, drinking alcohol, eating herbs, taking vitamin C, and receiving COVID-19 information were statistically significant (*p*-value < 0.05). These findings are presented in Table 1.

### 3.2. Participants’ Knowledge and Preventive Behaviors toward COVID-19

Table 2 presents a summary of the level of COVID-19 knowledge and preventive behaviors among an adult population of northern Thailand. The mean score of COVID-19 knowledge was 7.20 (SD = 0.93) and ranged from 5–9. The participants had moderate and low knowledge scores (65.5%, 24.4%), followed by high scores (10.1%). In addition, most of the vaccinated subjects had a moderately high score on COVID-19 knowledge (72.0%), while 15.0% and 13.0% had a high and low level of such knowledge, respectively. In terms of COVID-19 preventive behaviors, scores ranged from 24–43, with a mean of 33.0 (SD = 4.84). Nearly half of the participants had a moderate level of preventive behaviors (42.0%), while those with a high and low level of knowledge accounted for 39.0% and 19.0% of the sample, respectively. Interestingly, the preventive behaviors of the vaccinated participants had a good score (46.4%) followed by a moderate (41.6%), and low level (12.0%) of preventive behaviors, respectively. Additionally, an interesting variable found that there was a statistically significant difference in the scores of COVID-19 knowledge and preventive behaviors between those who were vaccinated and those who were not (*p*-value < 0.001).

### 3.3. Relationship between the Characteristics Data Variables with COVID-19 Knowledge and Preventive Behaviors

Table 3 presents an analysis of the relationship between the sample characteristics data variables with COVID-19 knowledge and preventive behaviors. Interesting variables found that age, education level, occupation, current disease, eating herbs, taking vitamin C, and receiving COVID-19 information were statistically related to COVID-19 knowledge (*p*-value < 0.05). There was a significant difference in COVID-19 preventive behavior scores between both sexes and disease groups (*p*-value < 0.05).

### 3.4. COVID-19 Knowledge Related to Preventive Behaviors and Vaccination

Overall, the score of COVID-19 knowledge was significantly correlated with the score of preventive behaviors (r = 0.516, *p* < 0.001). Binary logistical regression revealed that the score of COVID-19 knowledge was significantly associated with getting COVID-19 vaccines among the sample group after adjusting for age, education, occupation, financial status, and current disease (*p* < 0.001) (Table 4). The modeled odds of receiving a vaccine increased by 87% per unit increase in COVID-19 knowledge. For linear regression analysis, the final model showed that the COVID-19 preventive behavior score was related to the COVID-19 knowledge score, age, underlying disease, and BMI (*p* < 0.05) (Table 5). The increase in COVID-19 knowledge per point resulted in a 2.64-point decrease in COVID-19 behaviors.

## 4. Discussion

This study was conducted to study personal factors, COVID-19 knowledge and preventive behaviors, and vaccination among the general population living in the rural areas of northern Thailand between September and October 2021 during the third wave of COVID-19, when the rate of vaccination in rural regions was still low and the number of infected people was increasing in Thailand. The results show that the number of participants in the rural northern areas who were vaccinated was low (32.8%), implying that an appropriate implementation program to address the challenge of low rates of vaccination should be considered. This suggests that despite the government’s plan to provide COVID-19 vaccinations to the Thai population, many people from different areas of the country are unable to access the vaccine. This is similar to a previous study that showed the vaccination acceptance rate was different among the general population aged 18 years and older, based on a survey and literature review [1]. Further, hesitancy toward the COVID-19 vaccine has been documented among the general population in 11 countries. However, the World Health Organization has pushed for many countries to have strategic plans for vaccinating against COVID-19 (the global COVID-19 vaccination strategy) and making the vaccine available: there is an urgent agenda to accomplish this within tight deadlines [24]. For Thailand, it is very important for public relations to establish COVID-10 campaigns to encourage people to receive vaccines against COVID-19 in order to create herd immunity [25]. Therefore, support is necessary for governmental and non-governmental initiatives to procure and distribute the vaccine and provide people with quick access to effective vaccination services [25]. This is consistent with a study that found the reluctance to vaccinate among the Jordanian population was higher compared to most other populations only 36.8% of study participants had been vaccinated after the vaccine was made available [26]. Another study showed that 49% of respondents were willing to vaccinate, but 28% were undecided [27]. This was because the respondents were uncertain about the vaccine and its side effects, as well as the vaccines’ effectiveness and benefits [27].

In analyzing personal factor variables, we found that age, education, occupation, financial status, and current disease were significantly associated with vaccination against COVID-19 among people living in rural areas. Several studies have shown that socioeconomic factors, including having higher education and a decent income, are associated with increased willingness to receive a vaccine against COVID-19 [28,29,30]. Other studies have found that age ranges, education, and high income of individuals are predictors of acceptance of COVID-19 vaccines [31,32,33,34]. As the previous study explained, a person’s acceptance of vaccination varies according to individual factors such as age and the presence of underlying diseases [35]. The results of the study are consistent with previous meta-analysis studies, which found that morbidity, perceived risk, and severity of disease were statistically significant predictors of vaccination acceptance behavior [36,37]. This accords with a previous literature review which pointed out that COVID-19 and non-communicable diseases (NCDs) are closely related [38,39]. Non-communicable diseases, including cardiovascular disease (CVD), blood pressure, and diabetes, are significant predictors of severe morbidity and mortality from COVID-19 [38,39,40]. These individuals with non-communicable diseases should be highly aware of their vaccination decisions [38,39,40]. Protection Motivation Theory [41] states that if a person assesses the threats and is able to cope with them, such behavior leads to an adaptive response to disease prevention. Therefore, if a person senses that his/her health is at risk, the level of fear will increase, which will motivate that person to adopt self-defense behaviors [41,42]. A part of this may also be due to the Thai government’s policy that focuses on providing vaccination against COVID-19 among individuals at risk, such as the elderly and those with disease [15,25].

Interestingly, this study found that factors such as smoking, drinking alcohol, exercising, taking supplementary herbs and vitamin C, and COVID-19 preventive behaviors have a significant association with COVID-19 vaccines. This study found that people who practice good self-care behaviors are more likely to be vaccinated, and Thai herbs are used as an alternative medicine to support health in the Thai context in fighting COVID-19. The study found that self-care and healthy behaviors are motivated by the perceived risks of the disease and persuade individuals to seek information that benefits their health [41]. In line with literature reviews, this study found that exercise has had an impact on COVID vaccination decisions [43,44]. We found that respondents who used herbs and took vitamin C expected these health strategies to prevent COVID-19 infection [45]. One study has shown that the use of herbs or vitamin supplements helps boost antiviral potential against the SARS-CoV-2 virus and can protect against COVID-19. So too can supplements in the form of food intake or adjuvant therapy that prevents infection and strengthens immunity. However, the efficacy of this hypothesis requires further trial validation for patients with SARS-CoV-2 and COVID-19 [45]. Consistent with previous studies, the samples highlight the importance of consuming vitamin C on a daily and weekly basis to increase immunity against COVID-19 [46]. Compared to the pre-pandemic period with the pandemic, it has increased from 35.5% to 41%, which is statistically significant [46].

The most important factor in this study, Covid-19 knowledge, was positively associated with preventive behavior scores and vaccination against COVID-19. However, we found that overall, the numbers of those who favored vaccination were low. The questionnaire answers revealed poor knowledge of the ways COVID-19 is transmitted. For example, most cases of COVID-19, which is the same virus that causes severe acute respiratory syndrome (SARS), are spread through droplets produced by an infected person coughing, sneezing, or speaking. In addition, the respondents’ scores related to COVID-19 prevention were low, particularly with regard to the symptoms—sore throat, nausea and vomiting, fatigue, shortness of breath, and difficulty breathing. The study pointed out that a person’s experience through learning can change his/her behaviors toward health and self-care [47]. Furthermore, an individual’s learning process depends on internal factors such as experiences and beliefs affecting their behavior, which leads to preventive behaviors [41]. Consistent with several studies, COVID-19 disease risk perceptions, disease persistence, and severity are associated with higher COVID-19 vaccine acceptance [44,48,49]. Furthermore, these predictors can be used to understand the acceptance of the COVID-19 vaccine and the intention to receive vaccines among different population groups [1]. Another study has found that the Malaysian population has good knowledge of, and perceptions toward, preventing COVID-19, which is the main reason for respondents’ higher acceptance of the COVID-19 vaccine [50,51]. Additionally, a study from China found that health literacy and electronic health are important predictors of preventive health behaviors among Chinese [52,53]. In Bangladesh, people with higher knowledge scores reported higher COVID-19 preventive behaviors [54]. In summary, individuals who have a good understanding of COVID-19 may contribute to positive self-preventive behaviors and actions, as well as vaccine acceptance, which results in vaccination. The current study also showed that education level, taking vitamin C, and receiving COVID-19 information are positively associated with COVID-19 knowledge. A person’s learning ability arises from his/her experience or knowledge gained. This is consistent with the notion of Bandura [47], who mentioned that a person’s learning is caused by many components of the learning process, especially the person’s ability. A prior study found that access to COVID-19 information and awareness of prevention measures and their benefits increases the likelihood of participating in more COVID-19 prevention measures [55]. Interestingly, those who are elderly, unemployed, have a current disease, or use herbs have lower COVID-19 knowledge. This suggests that increasing knowledge should be emphasized in these groups.

Besides, the results of the univariate analysis reveal that there is a difference in COVID-19 preventive behaviors between both sexes. In the multivariable model, many factors, including age, BMI, and current disease are associated with COVID-19 preventive behaviors. This study found that most people in the rural areas demonstrated a moderate level of preventive behavior against COVID-19. However, 70% of those in the rural areas had a low level of knowledge about elementary preventive measures such as washing hands with alcohol gel when going out in public. This group also knew when they were sick with fever, were coughing, sneezing, or had a runny nose or sore throat, they should buy medicine and take it. This is consistent with previous studies, which found that females were more likely to engage in preventive health behaviors than men, and that age is a predictive variable in the study of preventive health behaviors [43,56]. The results are similar to a study that showed adults who are overweight or obese are significantly more likely to report washing or sanitizing their hands, and staying at home because they felt unwell, compared with adults who are of normal weight [57]. Similarly, a study by Hosen et al. [54] found that male participants with a high number of comorbidities reported lower preventive behaviors against COVID-19.

The strength of this study is that the variables are important for the development of educational awareness campaigns toward COVID-19 and vaccination among rural populations. However, the study has limitations: First, this study was conducted when COVID-19 vaccines were not widely available, so the rate of COVID-19 vaccination of the population now may be different to what it was during the study. Second, this study has not assessed other factors such as attitude and self-efficacy toward COVID-19. Therefore, further studies could consider these aspects in order to gain a deeper understanding of an individual’s attitudes toward COVID-19 prevention and control, and vaccination behaviors. Third, this research was conducted among the rural population living in northern Thailand, so the findings are not necessarily generalizable to other populations. Future qualitative studies should be considered to develop a model to promote health and advance the COVID-19 vaccination service to reduce the incidence and severity of the pandemic.

## 5. Conclusions

The findings of this study show that COVID-19 knowledge is associated with COVID-19 prevention behaviors and vaccination among a rural population in northern Thailand and that these factors are very important in preventing and controlling the disease. Enhancing the public’s knowledge of the disease and its health consequences will increase the likelihood that people will become more aware of the transmission of infectious disease and take effective precautions against COVID-19. In addition to broader public education campaigns about COVID-19, specific efforts should be made to facilitate healthcare patient–provider communication about COVID-19 vaccination, focusing on safety and efficacy. Therefore, developing actionable strategies to communicate the potential benefits of vaccination, safety, and efficacy can improve the vaccine acceptance rate and help accelerate efforts to contain this pandemic.

## Figures and Tables

**Table 1:** General characteristics of participants categorized by vaccine group (*n* = 1524).

Variable	All	No Vaccine	Vaccine	*p*-Value
Sex				0.112 ^a^
Male	672 (44.1%)	466 (45.5%)	206 (41.2%)	
Female	852 (55.9%)	558 (54.5%)	294 (58.8%)	
Age (years)				0.028 ^a^
<30	389 (25.5%)	267 (26.1%)	122 (24.4%)	
30–39	318 (20.9%)	201 (19.6%)	117 (23.4%)	
40–49	224 (14.7%)	137 (13.4%)	87 (17.4%)	
50–59	247 (16.2%)	181 (17.7%)	66 (13.2%)	
≥60	346 (22.7%)	238 (23.2%)	108 (21.6%)	
Mean ± SD	44.13 ± 16.25	44.45 ± 16.63	43.47 ± 15.45	
Min.–Max.	20–89	20–89	20–80	
Marital status				0.948 ^a^
Single/Widowed/Divorced/Separate	776 (50.9%)	522 (51.0%)	254 (50.8%)	
Married	748 (49.1%)	502 (49.0%)	246 (49.2%)	
Education				<0.001 ^a^
No	222 (14.6%)	170 (16.6%)	52 (10.4%)	
Primary school	457 (30.0%)	326 (31.8%)	131 (26.2%)	
Secondary school	473 (31.0%)	317 (31.0%)	156 (31.2%)	
Diploma/Bachelor degree	372 (24.4%)	211 (20.6%)	161 (32.2%)	
Occupation				<0.001 ^a^
No	212 (13.9%)	162 (15.8%)	50 (10.0%)	
Government/Private sector	199 (13.1%)	84 (8.2%)	115 (23.0%)	
Farmer	239 (15.7%)	157 (15.3%)	82 (16.4%)	
General employee	412 (27.0%)	302 (29.5%)	110 (22.0%)	
Merchant/Self-employed	260 (17.1%)	176 (17.2%)	84 (16.8%)	
Student	202 (13.3%)	143 (14.0%)	59 (11.8%)	
Financial status				<0.001 ^a^
Insufficient	582 (38.2%)	447 (43.7%)	135 (27.0%)	
Sufficient	942 (61.8%)	577 (56.3%)	365 (73.0%)	
BMI				0.060 ^a^
<18.5 g/m^2^	86 (5.6%)	61 (6.0%)	25 (5.0%)	
18.5–22.9 kg/m^2^	728 (47.8%)	509 (49.7%)	219 (43.8%)	
23.0–24.9 kg/m^2^	353 (23.2%)	232 (22.7%)	121 (24.2%)	
≥25.0 kg/m^2^	357 (23.4%)	222 (21.7%)	135 (27.0%)	
Current disease				0.004 ^a^
No	1055 (69.2%)	733 (71.6%)	322 (64.4%)	
Yes	469 (30.8%)	291 (28.4%)	178 (35.6%)	
Smoking				<0.001 ^a^
No	998 (65.5%)	632 (61.7%)	366 (73.2%)	
Yes	526 (34.5%)	392 (38.3%)	134 (26.8%)	
Drinking alcohol				0.019 ^a^
No	914 (60.0%)	593 (57.9%)	321 (64.2%)	
Yes	610 (40.0%)	431 (42.1%)	179 (35.8%)	
Exercise				0.001 ^a^
No	819 (53.7%)	581 (56.7%)	238 (47.6%)	
Yes	705 (46.3%)	443 (43.3%)	262 (52.4%)	
Eating herb				0.009 ^a^
No	1014 (66.5%)	704 (68.8%)	310 (62.0%)	
Yes	510 (33.5%)	320 (31.2%)	190 (38.0%)	
Eating vitamin C				0.001 ^a^
No	1312 (86.1%)	902 (88.1%)	410 (82.0%)	
Yes	212 (13.9%)	122 (11.9%)	90 (18.0%)	
Receiving COVID-19 information				<0.001 ^a^
No	490 (32.2%)	367 (35.8%)	123 (24.6%)	
Yes	1034 (67.8%)	657 (64.2%)	377 (75.4%)	

^a^ Chi-square-test.

**Table 2:** COVID-19 knowledge and preventive behaviors of participants categorized by vaccine group (*n* = 1524).

Variables	Total *n* (%)	No Vaccine *n* (%)	Vaccine *n* (%)	*p*-Value
COVID-19 knowledge (scores)				<0.001 ^a^
Low level (0–6 Scores)	372 (24.4)	307 (30.0)	65 (13.0)	
Moderate level (7–8 Scores)	998 (65.5)	638 (62.3)	360 (72.0)	
High level (9–12 Scores)	154 (10.1)	79 (7.7)	75 (15.0)	
Mean ± SD	7.20 ± 0.93	7.04 ± 0.90	7.54 ± 0.90	
Min.–Max.	5.00–9.00	5.00–9.00	5.00–9.00	
COVID-19 preventive behaviors (scores)				<0.001 ^a^
Low level (0–27 Scores)	290 (19.0)	230 (22.5)	60 (12.0)	
Moderate level (28–35 Scores)	640 (42.0)	432 (42.1)	208 (41.6)	
High level (36–45 Scores)	594 (39.0)	362 (35.4)	232 (46.4)	
Mean ± SD	33.00 ± 4.84	32.50 ± 4.97	34.02 ± 4.37	
Min.–Max.	24.00–43.00	24.00–43.00	25.00–43.00	

^a^ Independent *t*-test.

**Table 3:** COVID-19 knowledge and preventive behaviors scores classified according to the general characteristics of participants.

Variable	Knowledge		Behaviors	
	Mean ± SD	*p*-Value	Mean ± SD	*p*-Value
Sex		0.395 ^a^		0.042 ^a^
Male	7.18 ± 0.93		32.72 ± 4.83	
Female	7.22 ± 0.93		33.22 ± 4.83	
Age		<0.001 ^b^		0.151 ^b^
<30 years	7.29 ± 0.94		32.86 ± 4.88	
30–39 years	7.27 ± 0.89		32.85 ± 4.82	
40–49 years	7.31 ± 0.89		33.25 ± 4.66	
50–59 years	7.16 ± 0.92		33.62 ± 4.76	
≥60 years	6.99 ± 0.97		32.69 ± 4.94	
Marital status		0.521 ^a^		0.539 ^a^
Single/Widowed/Divorced/Separate	7.22 ± 0.93		33.07 ± 4.85	
Married	7.19 ± 0.94		32.92 ± 4.83	
Education		<0.001 ^b^		0.959 ^b^
No	7.09 ± 0.87		32.88 ± 4.81	
Primary school	7.07 ± 0.95		33.09 ± 4.86	
Secondary school	7.29 ± 0.93		32.98 ± 4.93	
Diploma/Bachelor degree	7.31 ± 0.93		32.98 ± 4.72	
Occupation		<0.001 ^b^		0.367 ^b^
No	6.96 ± 0.86		32.52 ± 4.95	
Government/Private sector	7.49 ± 0.92		33.53 ± 4.69	
Farmer	7.25 ± 0.94		32.88 ± 4.75	
General employee	7.15 ± 0.95		33.13 ± 4.75	
Merchant/Self-employed	7.15 ± 0.88		33.07 ± 5.06	
Student	7.29 ± 0.96		32.77 ± 4.83	
Financial status		0.138 ^a^		0.235 ^a^
Insufficient	7.16 ± 0.91		33.19 ± 4.91	
Sufficient	7.23 ± 0.95		32.88 ± 4.79	
BMI		0.905 ^b^		0.094 ^b^
<18.5 g/m^2^	7.16 ± 0.93		33.13 ± 4.91	
18.5–22.9 kg/m^2^	7.19 ± 0.94		32.68 ± 4.82	
23.0–24.9 kg/m^2^	7.23 ± 0.91		33.27 ± 4.68	
≥25.0 kg/m^2^	7.20 ± 0.95		33.36 ± 4.98	
Current disease		<0.001 ^a^		<0.001 ^a^
No	7.31 ± 0.91		33.43 ± 4.64	
Yes	6.97 ± 0.95		32.03 ± 5.13	
Smoking		0.775 ^a^		0.789 ^a^
No	7.21 ± 0.94		32.98 ± 4.84	
Yes	7.19 ± 0.91		33.05 ± 4.83	
Drinking alcohol		0.571 ^a^		0.705 ^a^
No	7.19 ± 0.92		32.96 ± 4.89	
Yes	7.22 ± 0.95		33.06 ± 4.76	
Exercise		0.999 ^a^		0.279 ^a^
No	7.20 ± 0.93		32.88 ± 4.90	
Yes	7.20 ± 0.93		33.14 ± 4.76	
Eating herb		0.009 ^a^		0.221 ^a^
No	7.25 ± 0.93		33.11 ± 4.87	
Yes	7.11 ± 0.93		32.79 ± 4.77	
Eating vitamin C		0.013 ^a^		0.771 ^a^
No	7.18 ± 0.93		32.99 ± 4.84	
Yes	7.35 ± 0.93		33.09 ± 4.82	
Receiving COVID-19 information		0.001 ^a^		0.540 ^a^
No	7.09 ± 0.90		32.89 ± 4.92	
Yes	7.25 ± 0.94		33.05 ± 4.80	

^a^ Independent *t*-test. ^b^ One-way ANOVA.

**Table 4:** Factors associated with getting COVID-19 vaccine among participants by binary logistic regression.

Factor	B	S.E.	*p*-Value	OR	95% CI
Age					
<30 years	Ref.		0.011	1	
30–39 years	0.355	0.177	0.045	1.427	1.008, 2.019
40–49 years	0.567	0.208	0.006	1.762	1.172, 2.649
50–59 years	0.282	0.243	0.247	1.326	0.823, 2.135
≥60 years	0.722	0.248	0.004	2.059	1.267, 3.346
Education					
No	Ref.		0.001	1	
Primary school	0.063	0.212	0.766	1.065	0.703, 1.615
Secondary school	0.448	0.256	0.080	1.565	0.948–2.583
Diploma/Bachelor degree	0.938	0.275	0.001	2.555	1.492, 4.376
Occupation					
No	Ref.		<0.001	1	
Government/Private sector	1.097	0.252	<0.001	2.996	1.828, 4.909
Farmer	0.368	0.231	0.111	1.445	0.919, 2.272
General employee	0.248	0.219	0.258	1.282	0.834, 1.969
Merchant/Self-employed	0.332	0.238	0.162	1.394	0.875, 2.221
Student	0.034	0.269	0.898	1.035	0.611, 1.755
Financial status (sufficient)	0.634	0.134	<0.001	1.885	1.450, 2.451
Current disease (yes)	0.769	0.142	<0.001	2.157	1.633, 2.848
COVID-19 knowledge (scores)	0.626	0.066	<0.001	1.870	1.643, 2.128

B = regression coefficient; S.E. = standard error; OR = odds ratio; 95%CI = 95% confidence interval; Ref. = reference group.

**Table 5:** Factors associated with a score of COVID-19 preventive behaviors about among participants by multiple linear regression.

Factor	B	S.E.	Beta	*p*-Value	95% CI
Age					
<30 years	Ref.				
30–39 years	0.010	0.311	0.001	0.975	−0.601, 0.620
40–49 years	0.383	0.346	0.028	0.269	−0.296, 1.062
50–59 years	1.305	0.342	0.099	<0.001	0.634, 1.975
≥60 years	0.942	0.321	0.082	0.003	0.311, 1.572
Current disease (yes)	−0.983	0.251	−0.094	<0.001	−1.475, −0.491
BMI					
<18.5 g/m^2^	0.455	0.469	0.022	0.333	−0.466, 1.375
18.5–22.9 kg/m^2^	Ref.				
23.0–24.9 kg/m^2^	0.516	0.268	0.045	0.054	−0.009, 1.042
≥25.0 kg/m^2^	0.746	0.271	0.065	0.006	0.214, 1.278
COVID-19 knowledge (scores)	2.644	0.115	0.510	<0.001	2.419, 2.870

B = Unstandardized coefficients; S.E. = standard error; Beta = standardized coefficients; 95% CI = 95% confidence interval; Ref. = reference group.

## Data Availability

The datasets used and/or analyzed during the current study are available from the corresponding author on reasonable request.
